# Joint effects of residential greenness and genetic susceptibility on type 2 diabetes: a prospective cohort study using satellite-derived Normalized Difference Vegetation Index

**DOI:** 10.1093/lifemeta/loag019

**Published:** 2026-07-08

**Authors:** Yonghua Yu, Junru Fan, Qiuyu Cao, Dong Li, Hong Lin, Zhen Ye, Ruizhi Zheng, Yu Xu, Min Xu, Mian Li, Libin Zhou, Shuangyuan Wang, Tiange Wang, Zhiyun Zhao, Jie Zheng, Guang Ning, Weiqing Wang, Ruying Hu, Yufang Bi, Jieli Lu

**Affiliations:** Department of Endocrine and Metabolic Diseases, Shanghai Institute of Endocrine and Metabolic Diseases, Ruijin Hospital, Shanghai Jiao Tong University School of Medicine, Shanghai 200025, China; Shanghai National Clinical Research Center for Endocrine and Metabolic Diseases, Key Laboratory for Endocrine and Metabolic Diseases of the National Health Commission of the P.R. China, Shanghai National Center for Translational Medicine, Shanghai Digital Medicine Innovation Center, Ruijin Hospital, Shanghai Jiao Tong University School of Medicine, Shanghai 200025, China; Department of Endocrine and Metabolic Diseases, Shanghai Institute of Endocrine and Metabolic Diseases, Ruijin Hospital, Shanghai Jiao Tong University School of Medicine, Shanghai 200025, China; Shanghai National Clinical Research Center for Endocrine and Metabolic Diseases, Key Laboratory for Endocrine and Metabolic Diseases of the National Health Commission of the P.R. China, Shanghai National Center for Translational Medicine, Shanghai Digital Medicine Innovation Center, Ruijin Hospital, Shanghai Jiao Tong University School of Medicine, Shanghai 200025, China; Department of Endocrine and Metabolic Diseases, Shanghai Institute of Endocrine and Metabolic Diseases, Ruijin Hospital, Shanghai Jiao Tong University School of Medicine, Shanghai 200025, China; Shanghai National Clinical Research Center for Endocrine and Metabolic Diseases, Key Laboratory for Endocrine and Metabolic Diseases of the National Health Commission of the P.R. China, Shanghai National Center for Translational Medicine, Shanghai Digital Medicine Innovation Center, Ruijin Hospital, Shanghai Jiao Tong University School of Medicine, Shanghai 200025, China; Institute for Urban Governance and Sustainable Development, Tsinghua University, Beijing 100084, China; Department of Endocrine and Metabolic Diseases, Shanghai Institute of Endocrine and Metabolic Diseases, Ruijin Hospital, Shanghai Jiao Tong University School of Medicine, Shanghai 200025, China; Shanghai National Clinical Research Center for Endocrine and Metabolic Diseases, Key Laboratory for Endocrine and Metabolic Diseases of the National Health Commission of the P.R. China, Shanghai National Center for Translational Medicine, Shanghai Digital Medicine Innovation Center, Ruijin Hospital, Shanghai Jiao Tong University School of Medicine, Shanghai 200025, China; Zhejiang Provincial Center for Disease Control and Prevention, Hangzhou, Zhejiang 310051, China; Department of Endocrine and Metabolic Diseases, Shanghai Institute of Endocrine and Metabolic Diseases, Ruijin Hospital, Shanghai Jiao Tong University School of Medicine, Shanghai 200025, China; Shanghai National Clinical Research Center for Endocrine and Metabolic Diseases, Key Laboratory for Endocrine and Metabolic Diseases of the National Health Commission of the P.R. China, Shanghai National Center for Translational Medicine, Shanghai Digital Medicine Innovation Center, Ruijin Hospital, Shanghai Jiao Tong University School of Medicine, Shanghai 200025, China; Department of Endocrine and Metabolic Diseases, Shanghai Institute of Endocrine and Metabolic Diseases, Ruijin Hospital, Shanghai Jiao Tong University School of Medicine, Shanghai 200025, China; Shanghai National Clinical Research Center for Endocrine and Metabolic Diseases, Key Laboratory for Endocrine and Metabolic Diseases of the National Health Commission of the P.R. China, Shanghai National Center for Translational Medicine, Shanghai Digital Medicine Innovation Center, Ruijin Hospital, Shanghai Jiao Tong University School of Medicine, Shanghai 200025, China; Department of Endocrine and Metabolic Diseases, Shanghai Institute of Endocrine and Metabolic Diseases, Ruijin Hospital, Shanghai Jiao Tong University School of Medicine, Shanghai 200025, China; Shanghai National Clinical Research Center for Endocrine and Metabolic Diseases, Key Laboratory for Endocrine and Metabolic Diseases of the National Health Commission of the P.R. China, Shanghai National Center for Translational Medicine, Shanghai Digital Medicine Innovation Center, Ruijin Hospital, Shanghai Jiao Tong University School of Medicine, Shanghai 200025, China; Department of Endocrine and Metabolic Diseases, Shanghai Institute of Endocrine and Metabolic Diseases, Ruijin Hospital, Shanghai Jiao Tong University School of Medicine, Shanghai 200025, China; Shanghai National Clinical Research Center for Endocrine and Metabolic Diseases, Key Laboratory for Endocrine and Metabolic Diseases of the National Health Commission of the P.R. China, Shanghai National Center for Translational Medicine, Shanghai Digital Medicine Innovation Center, Ruijin Hospital, Shanghai Jiao Tong University School of Medicine, Shanghai 200025, China; Department of Endocrine and Metabolic Diseases, Shanghai Institute of Endocrine and Metabolic Diseases, Ruijin Hospital, Shanghai Jiao Tong University School of Medicine, Shanghai 200025, China; Shanghai National Clinical Research Center for Endocrine and Metabolic Diseases, Key Laboratory for Endocrine and Metabolic Diseases of the National Health Commission of the P.R. China, Shanghai National Center for Translational Medicine, Shanghai Digital Medicine Innovation Center, Ruijin Hospital, Shanghai Jiao Tong University School of Medicine, Shanghai 200025, China; Department of Endocrine and Metabolic Diseases, Shanghai Institute of Endocrine and Metabolic Diseases, Ruijin Hospital, Shanghai Jiao Tong University School of Medicine, Shanghai 200025, China; Shanghai National Clinical Research Center for Endocrine and Metabolic Diseases, Key Laboratory for Endocrine and Metabolic Diseases of the National Health Commission of the P.R. China, Shanghai National Center for Translational Medicine, Shanghai Digital Medicine Innovation Center, Ruijin Hospital, Shanghai Jiao Tong University School of Medicine, Shanghai 200025, China; Department of Endocrine and Metabolic Diseases, Shanghai Institute of Endocrine and Metabolic Diseases, Ruijin Hospital, Shanghai Jiao Tong University School of Medicine, Shanghai 200025, China; Shanghai National Clinical Research Center for Endocrine and Metabolic Diseases, Key Laboratory for Endocrine and Metabolic Diseases of the National Health Commission of the P.R. China, Shanghai National Center for Translational Medicine, Shanghai Digital Medicine Innovation Center, Ruijin Hospital, Shanghai Jiao Tong University School of Medicine, Shanghai 200025, China; Department of Endocrine and Metabolic Diseases, Shanghai Institute of Endocrine and Metabolic Diseases, Ruijin Hospital, Shanghai Jiao Tong University School of Medicine, Shanghai 200025, China; Shanghai National Clinical Research Center for Endocrine and Metabolic Diseases, Key Laboratory for Endocrine and Metabolic Diseases of the National Health Commission of the P.R. China, Shanghai National Center for Translational Medicine, Shanghai Digital Medicine Innovation Center, Ruijin Hospital, Shanghai Jiao Tong University School of Medicine, Shanghai 200025, China; Department of Endocrine and Metabolic Diseases, Shanghai Institute of Endocrine and Metabolic Diseases, Ruijin Hospital, Shanghai Jiao Tong University School of Medicine, Shanghai 200025, China; Shanghai National Clinical Research Center for Endocrine and Metabolic Diseases, Key Laboratory for Endocrine and Metabolic Diseases of the National Health Commission of the P.R. China, Shanghai National Center for Translational Medicine, Shanghai Digital Medicine Innovation Center, Ruijin Hospital, Shanghai Jiao Tong University School of Medicine, Shanghai 200025, China; Department of Endocrine and Metabolic Diseases, Shanghai Institute of Endocrine and Metabolic Diseases, Ruijin Hospital, Shanghai Jiao Tong University School of Medicine, Shanghai 200025, China; Shanghai National Clinical Research Center for Endocrine and Metabolic Diseases, Key Laboratory for Endocrine and Metabolic Diseases of the National Health Commission of the P.R. China, Shanghai National Center for Translational Medicine, Shanghai Digital Medicine Innovation Center, Ruijin Hospital, Shanghai Jiao Tong University School of Medicine, Shanghai 200025, China; Department of Endocrine and Metabolic Diseases, Shanghai Institute of Endocrine and Metabolic Diseases, Ruijin Hospital, Shanghai Jiao Tong University School of Medicine, Shanghai 200025, China; Shanghai National Clinical Research Center for Endocrine and Metabolic Diseases, Key Laboratory for Endocrine and Metabolic Diseases of the National Health Commission of the P.R. China, Shanghai National Center for Translational Medicine, Shanghai Digital Medicine Innovation Center, Ruijin Hospital, Shanghai Jiao Tong University School of Medicine, Shanghai 200025, China; Zhejiang Provincial Center for Disease Control and Prevention, Hangzhou, Zhejiang 310051, China; Department of Endocrine and Metabolic Diseases, Shanghai Institute of Endocrine and Metabolic Diseases, Ruijin Hospital, Shanghai Jiao Tong University School of Medicine, Shanghai 200025, China; Shanghai National Clinical Research Center for Endocrine and Metabolic Diseases, Key Laboratory for Endocrine and Metabolic Diseases of the National Health Commission of the P.R. China, Shanghai National Center for Translational Medicine, Shanghai Digital Medicine Innovation Center, Ruijin Hospital, Shanghai Jiao Tong University School of Medicine, Shanghai 200025, China; Department of Endocrine and Metabolic Diseases, Shanghai Institute of Endocrine and Metabolic Diseases, Ruijin Hospital, Shanghai Jiao Tong University School of Medicine, Shanghai 200025, China; Shanghai National Clinical Research Center for Endocrine and Metabolic Diseases, Key Laboratory for Endocrine and Metabolic Diseases of the National Health Commission of the P.R. China, Shanghai National Center for Translational Medicine, Shanghai Digital Medicine Innovation Center, Ruijin Hospital, Shanghai Jiao Tong University School of Medicine, Shanghai 200025, China

**Keywords:** residential greenness, genetic susceptibility, type 2 diabetes, gene-environment interaction, Normalized Difference Vegetation Index (NDVI)

## Abstract

Residential greenness has been linked to a reduced risk of type 2 diabetes (T2D), yet whether genetic susceptibility modifies this association remains unclear. Evidence on gene-environment interaction is limited, mainly cross-sectional, and largely from European-ancestry populations. Prospective data from Chinese populations examining the association between residential greenness and incident T2D, as well as glycemic traits, are scarce. In this study, we included 7861 middle-aged and older Chinese adults (aged ≥ 40 years), and assessed residential greenness using the Normalized Difference Vegetation Index (NDVI) derived from Moderate-Resolution Imaging Spectroradiometer (MODIS) satellite imagery, linking it to T2D incidence over a median follow-up of 3.8 years. Genetic susceptibility was assessed in 5389 participants with DNA data using a T2D-specific weighted genetic risk score based on 89 genome-wide significant single-nucleotide polymorphisms identified in East Asian populations, weighted by published effect estimates. Our results show that higher residential greenness was associated with a 44% reduction in T2D risk (hazard ratio [HR] = 0.56, 95% confidence interval [CI]: 0.48–0.66), as well as improved insulin sensitivity and β-cell function. Notably, genetic susceptibility significantly modified the association between residential greenness and T2D on both multiplicative (*P* for interaction = 0.019) and additive scales (relative excess risk due to interaction [RERI] = 0.19), with higher greenness conferring greater relative and absolute risk reductions among individuals at medium and high genetic risk. These findings underscore a protective role of residential greenness against T2D, with stronger effects in those at higher genetic risk, supporting environmental interventions to mitigate genetic predisposition in the prevention of T2D.

## Introduction

Type 2 diabetes (T2D) represents a growing global public health burden, with an estimated 589 million adults affected in 2024 and projections indicating an increase to 853 million by 2050 [1]. These alarming statistics reflect the hidden burden of the disease, and underscore the urgent need for risk assessment based on high-quality outcome measures.

The development of T2D is not driven by a single factor, but rather results from the long-term interplay between genetic susceptibility and environmental exposures [2]. Among environmental determinants, residential greenness has recently attracted attention for its potential protective effects against T2D [3–11]. Residential greenness can be objectively quantified using the Normalized Difference Vegetation Index (NDVI) derived from the Moderate-Resolution Imaging Spectroradiometer (MODIS) aboard the Terra satellite. Satellite-derived NDVI provides a spatially continuous and standardized measure of natural vegetation surrounding residential locations, enabling precise assessment of individual-level exposure to green environments [12]. However, most prospective evidence comes from Western populations, whereas studies in Chinese populations remain extremely limited and are often restricted to single regions or specific age groups [6, 13], limiting the generalizability of the findings to the broader population.

In addition, whether genetic susceptibility modifies the association between residential greenness and T2D risk is largely unknown. To date, only the UK Biobank study has investigated this interaction on a multiplicative scale, suggesting that greenness exposure may provide stronger protection against T2D prevalence among individuals with lower genetic risk [14]. However, additive interactions, which carry greater public health signifi­cance by indicating whether an environmental intervention yields larger absolute effects in specific subgroups, were not assessed [15]. Moreover, the cross-sectional design, reliance on self-reported diabetes diagnoses, and potential for misclassification bias restrict causal inference and underscore the need for large-scale, longitudinal studies incorporating objective glycemic assessments to clarify the potential protective effects of greenness across different genetic risk strata.

To address these gaps, we conducted a prospective cohort study among middle-aged and older adults in China. We aimed to systematically evaluate the associations between long-term resi­dential greenness, as assessed using MODIS-derived NDVI, and glucose homeostasis, as well as incident T2D. We further exa­mined whether genetic susceptibility modifies these associations on both multiplicative and additive scales, thereby providing new insights into how environmental exposures and genetic susceptibility jointly influence diabetes risk in an East Asian population.

## Results

### Baseline characteristics of the cohort and residential greenness (NDVI) distribution

Table 1 presents the baseline characteristics of the 7861 participants included in the final analysis. The mean baseline age was 52.17 (standard deviation [SD] = 7.84) years, and 58.8% were women. The mean body mass index (BMI) was 24.02 (SD = 3.12) kg/m^2^. Mean homeostasis model assessment of insulin resistance (HOMA-IR) and homeostasis model assessment of β-cell function (HOMA-B) values were 1.50 (SD = 0.95) and 63.52 (SD = 36.32), respectively, and the mean T2D-specific genetic risk score (GRS) was 5.95 (SD = 0.45). Overall, baseline characteristics were similar across tertiles of residential greenness, except that participants in the highest tertile reported higher levels of phy­sical activity.

**Table 1:** Participant characteristics overall and by tertile of baseline NDVI in 2011.^a^

Characteristics	Overall	NDVI T1	NDVI T2	NDVI T3
**Number of participants**	7861	2619	2616	2626
**NDVI**	0.37 (0.07)	0.30 (0.03)	0.36 (0.02)	0.45 (0.03)
**Age (year)**	52.17 (7.84)	51.41 (7.70)	52.89 (8.12)	52.22 (7.64)
**Female (%)**	4620 (58.80)	1542 (58.90)	1523 (58.20)	1555 (59.20)
**Education level (%)**				
** Primary school or less**	4608 (58.80)	1455 (55.70)	1594 (61.20)	1559 (59.60)
** Middle school**	2749 (35.10)	987 (37.80)	839 (32.20)	923 (35.30)
** High school or above**	478 (6.10)	172 (6.60)	171 (6.60)	135 (5.20)
**Smoking status (%)**				
** Never**	5515 (70.60)	1840 (70.70)	1813 (69.80)	1862 (71.30)
** Former**	248 (3.20)	81 (3.10)	91 (3.50)	76 (2.90)
** Current**	2048 (26.20)	682 (26.20)	693 (26.70)	673 (25.80)
**Alcohol consumption (%)**				
** Never**	6105 (78.50)	2045 (78.50)	2026 (78.50)	2034 (78.60)
** Former**	128 (1.60)	49 (1.90)	37 (1.40)	42 (1.60)
** Current**	1541 (19.80)	512 (19.60)	517 (20.00)	512 (19.80)
**Diet score**	1.73 (0.72)	1.79 (0.73)	1.74 (0.72)	1.65 (0.70)
**High physical activity (%)**	2547 (32.40)	688 (26.30)	894 (34.20)	965 (36.80)
**Sleep duration (h per night)**	8.17 (1.20)	8.01 (1.17)	8.23 (1.21)	8.26 (1.20)
**Body mass index (kg/m^2^)**	24.02 (3.12)	24.04 (3.31)	23.98 (3.02)	24.05 (3.03)
**Systolic blood pressure (mmHg)**	126.67 (17.78)	125.43 (17.79)	127.82 (18.31)	126.77 (17.14)
**Diastolic blood pressure (mmHg)**	77.56 (9.99)	77.31 (9.79)	77.88 (10.26)	77.47 (9.90)
**Fasting blood glucose (mmol/L)**	5.48 (0.48)	5.44 (0.48)	5.48 (0.47)	5.53 (0.47)
**2-h postload glucose (mmol/L)**	6.61 (1.61)	6.62 (1.64)	6.59 (1.63)	6.61 (1.56)
**Hemoglobin A1c (%)**	5.65 (0.37)	5.59 (0.38)	5.69 (0.37)	5.67 (0.35)
**HOMA-IR**	1.50 (0.95)	1.55 (0.89)	1.47 (0.82)	1.49 (1.12)
**HOMA-B**	63.52 (36.32)	67.44 (35.30)	62.42 (33.87)	60.69 (39.24)
**Low-density lipoprotein cholesterol (mmol/L)**	2.36 (0.71)	2.46 (0.71)	2.33 (0.69)	2.30 (0.71)
**Triglyceride (mmol/L)**	1.50 (1.14)	1.57 (1.20)	1.47 (1.12)	1.47 (1.11)
**High-density lipoprotein cholesterol (mmol/L)**	1.22 (0.31)	1.26 (0.31)	1.21 (0.31)	1.19 (0.32)
**Type 2 diabetes GRS**	5.95 (0.45)	5.96 (0.44)	5.94 (0.46)	5.94 (0.45)

a Values are mean (SD) for continuous variables, and *n* (%) for categorical variables.

**Figure 1:**
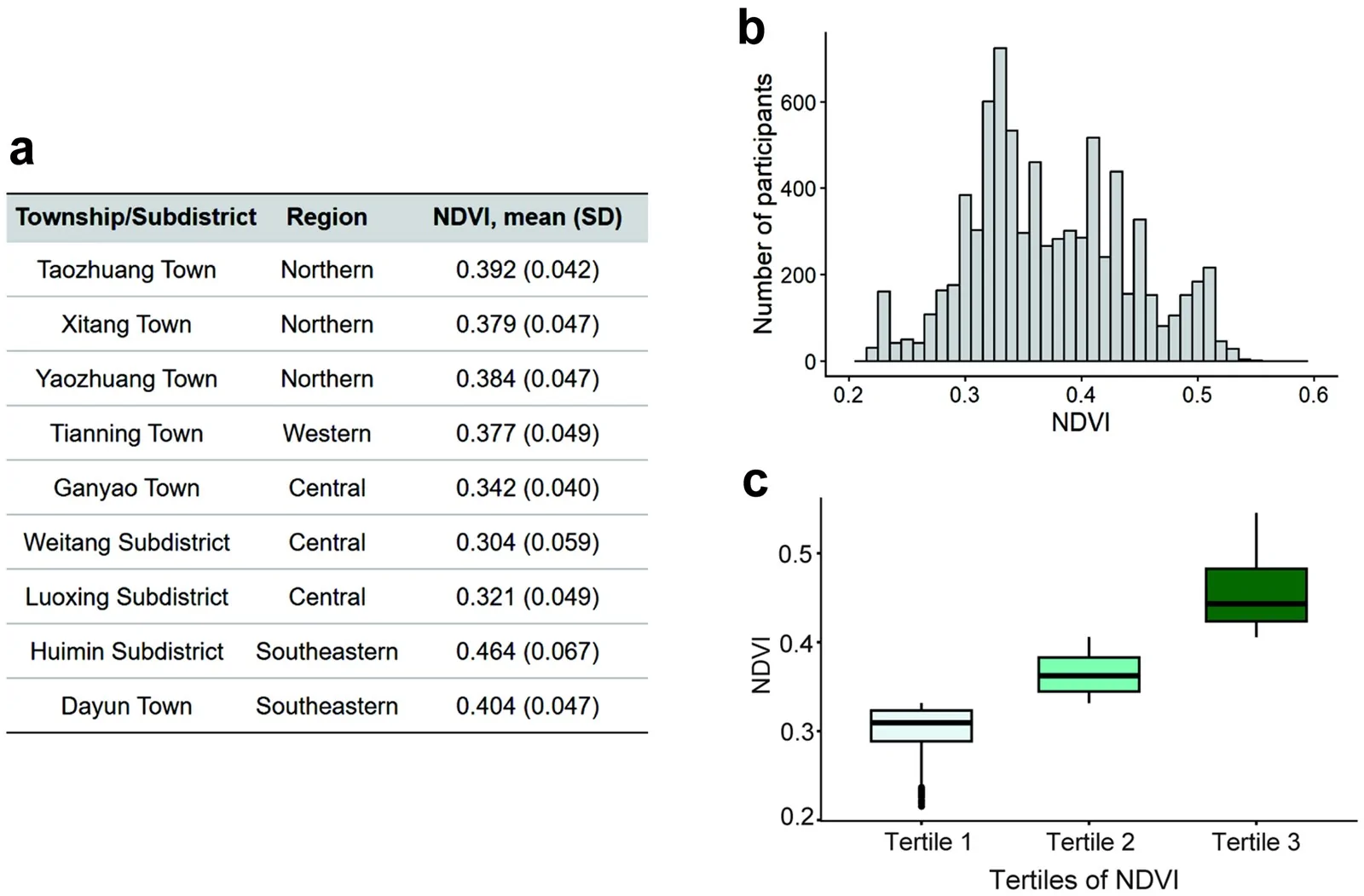
Distribution of residential greenness in Jiashan County, China (2011). (a) NDVI in Jiashan County, China, 2011. (b) Number of participants living in each level of residential NDVI. (c) Distribution of NDVI categorized by tertiles.

The spatial distribution of baseline residential greenness in Jiashan County, as indicated by satellite-derived NDVI values, is illustrated in Fig. 1. As shown in Fig. 1a, higher levels of residential greenness were concentrated in the southeastern (Dayun Town and Huimin Subdistrict) and northern (Taozhuang, Xitang and Yaozhuang Towns) regions, where mean NDVI values reached the 0.4–0.5 range. In contrast, the central regions exhibited rela­tively lower residential greenness, with mean NDVI values around 0.3. Most of the participants resided in areas with NDVI values between 0.3 and 0.4 (Fig. 1b). Across tertiles, NDVI values increased progressively, with the higher tertiles characterized by elevated medians and broader distributions (Fig. 1c), reflecting variations in participants’ exposure to residential greenness.

### Residential greenness is associated with glucose homeostasis and incident T2D

During a median follow-up of 3.8 years, 499 incident cases of T2D were documented. After adjustment for age and sex, higher resi­dential greenness was significantly associated with a lower risk of incident T2D (hazard ratio [HR] = 0.67, 95% confidence interval [CI]: 0.58–0.78 per 0.1-unit increase; Table 2, Model 1). This association remained robust after further adjustment for socioeconomic, behavioral, and metabolic factors. Additional adjustment for air pollution (time-varying particulate matter with an aerodynamic diameter ≤ 2.5 μm [PM_2.5_]) strengthened the inverse association between residential greenness and T2D risk (HR = 0.56, 95% CI: 0.48–0.66). Similar patterns were observed when residential greenness was categorized into tertiles. Compared with participants in the lowest tertile, those in the highest tertile had a substantially reduced risk of incident T2D (HR = 0.49, 95% CI: 0.38–0.63). In models with 250-m and 1250-m NDVI buffers, we also observed an inverse association between residential greenness and incident T2D (Supplementary Tables S1 and S2).

**Table 2:** Association between residential greenness and incident T2D.^a^

Model	HR (95% CI) for 0.1-unit increase in residential greenness	HR (95% CI) in tertiles of residential greenness			*P* for trend
		Tertile 1	Tertile 2	Tertile 3	
**Model 1**	0.67 (0.58–0.78)	1.00 (Ref)	0.69 (0.56–0.85)	0.56 (0.45–0.70)	< 0.0001
**Model 2**	0.68 (0.59–0.79)	1.00 (Ref)	0.71 (0.57–0.88)	0.57 (0.45–0.72)	< 0.0001
**Model 3**	0.68 (0.59–0.79)	1.00 (Ref)	0.71 (0.57–0.88)	0.58 (0.46–0.73)	< 0.0001
**Model 4**	0.56 (0.48–0.66)	1.00 (Ref)	0.71 (0.57–0.88)	0.49 (0.38–0.63)	< 0.0001

a Model 1: adjusted for age and sex. Model 2: Model 1 + education level, smoking status, alcohol drinking, physical activity, and healthy diet score. Model 3: Model 2 + body mass index, systolic blood pressure, total cholesterol, triglyceride, and high-density lipoprotein cholesterol. Model 4: Model 3 + time-varying PM_2.5_.

**Figure 2:**
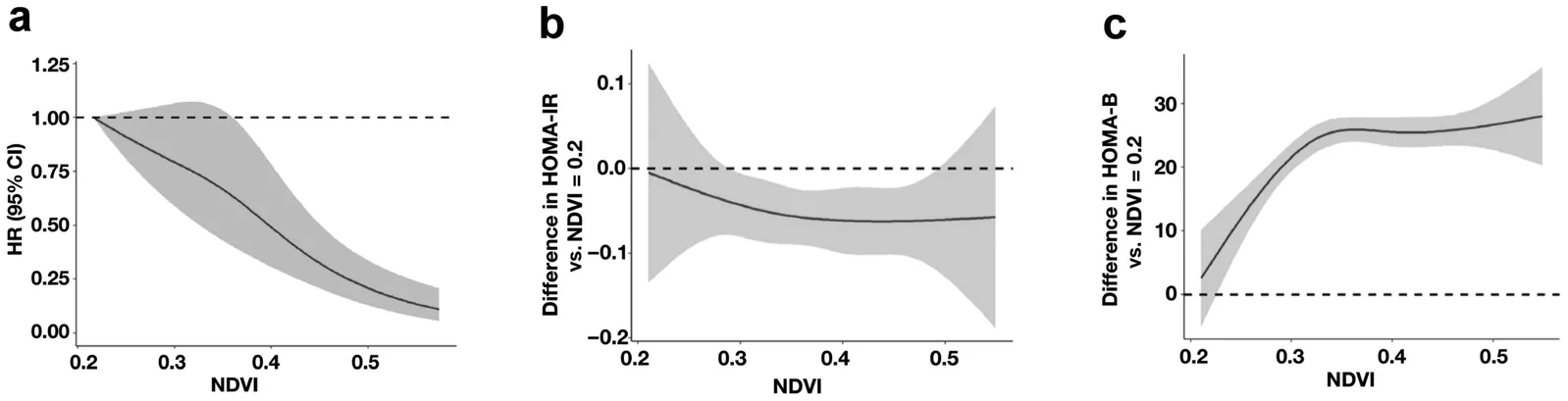
Dose-response relationships between residential greenness and incident T2D, as well as glucose homeostasis markers. (a) RCS curves for NDVI and incident T2D. (b) Difference in HOMA-IR vs. NDVI = 0.2. (c) Difference in HOMA-B vs. NDVI = 0.2. NDVI was fitted as a smooth term using an RCS with three knots. Shading areas indicate 95% CIs. Models were adjusted for age, sex, education level, smoking status, alcohol drinking, physical activity, healthy diet score, body mass index, systolic blood pressure, total cholesterol, triglyceride, high-density lipoprotein cholesterol, and time-varying PM_2.5_.

Restricted cubic spline (RCS) analyses showed the dose−response relationships between residential greenness and incident T2D, as well as glucose homeostasis indices (HOMA-IR and HOMA-B) (Fig. 2). After full adjustment for covariates, the HRs for incident T2D decreased monotonically with increasing NDVI, using 0.2 as the reference value, which was the lowest observed NDVI value (Fig. 2a). Residential greenness was inversely associated with HOMA-IR, indicating improved insulin sensitivity with higher NDVI levels (Fig. 2b). In contrast, the association between residential greenness and HOMA-B was non-linear, characterized by a sharp increase in HOMA-B up to an NDVI threshold of approximately 0.35, beyond which no further improvement was observed (Fig. 2c).

Stratified analyses further demonstrated a consistent, inverse dose-response association between residential greenness and incident T2D across all subgroups. This protective association persisted across categories of sex, age, smoking status, drinking status, and BMI (all *P* for trend < 0.05; Table 3; Supplementary Tables S3 and S4). Furthermore, no significant effect modification was observed for any of these factors (all *P* for interaction > 0.05).

**Table 3:** Stratified analysis on associations of greenness with incident T2D.^a^

Subgroups	HR (95% CI) in tertiles of residential greenness			*P* for trend	*P* for interaction
	Tertile 1	Tertile 2	Tertile 3		
**Sex**					0.495
** Male**	1.00 (Ref)	0.60 (0.45–0.82)	0.45 (0.32–0.63)	< 0.001	
** Female**	1.00 (Ref)	0.86 (0.63–1.18)	0.55 (0.38–0.79)	0.001	
**Age**					0.713
** < 55 years**	1.00 (Ref)	0.66 (0.49–0.89)	0.54 (0.39–0.74)	< 0.001	
** ≥ 55 years**	1.00 (Ref)	0.78 (0.57–1.08)	0.45 (0.31–0.65)	< 0.001	
**Current smoking**					0.450
** No**	1.00 (Ref)	0.72 (0.55–0.94)	0.54 (0.40–0.73)	< 0.001	
** Yes**	1.00 (Ref)	0.69 (0.48–1.00)	0.41 (0.26–0.63)	< 0.001	
**Current drinking**					0.284
** No**	1.00 (Ref)	0.73 (0.57–0.95)	0.56 (0.42–0.74)	< 0.001	
** Yes**	1.00 (Ref)	0.64 (0.43–0.97)	0.34 (0.21–0.56)	< 0.001	
**Physical activity**					0.603
** Inactive**	1.00 (Ref)	0.69 (0.55–0.87)	0.50 (0.39–0.65)	< 0.001	
** Active**	1.00 (Ref)	0.88 (0.47–1.64)	0.47 (0.21–1.07)	0.076	
**Body mass index**					0.295
** < 28 kg/m^2^**	1.00 (Ref)	0.68 (0.54–0.86)	0.49 (0.38–0.64)	< 0.001	
** ≥ 28 kg/m^2^**	1.00 (Ref)	0.85 (0.49–1.49)	0.44 (0.23–0.85)	0.013	

a Models are adjusted for age, sex, education level, smoking status, alcohol drinking, physical activity, healthy diet score, body mass index, systolic blood pressure, total cholesterol, triglyceride, high-density lipoprotein cholesterol, and time-varying PM_2.5_.

### Genetic predisposition modifies the association of residential greenness and T2D

We next examined whether genetic susceptibility to T2D modifies the association between residential greenness and incident T2D by stratifying participants according to a T2D-specific GRS. Higher GRS was associated with an increased risk of incident T2D, with HR of 1.15 (95% CI: 0.86–1.54) for the medium genetic risk group and 1.44 (95% CI: 1.09–1.91) for the high genetic risk group, compared with the low genetic risk group (Supplementary Table S5).

**Figure 3:**
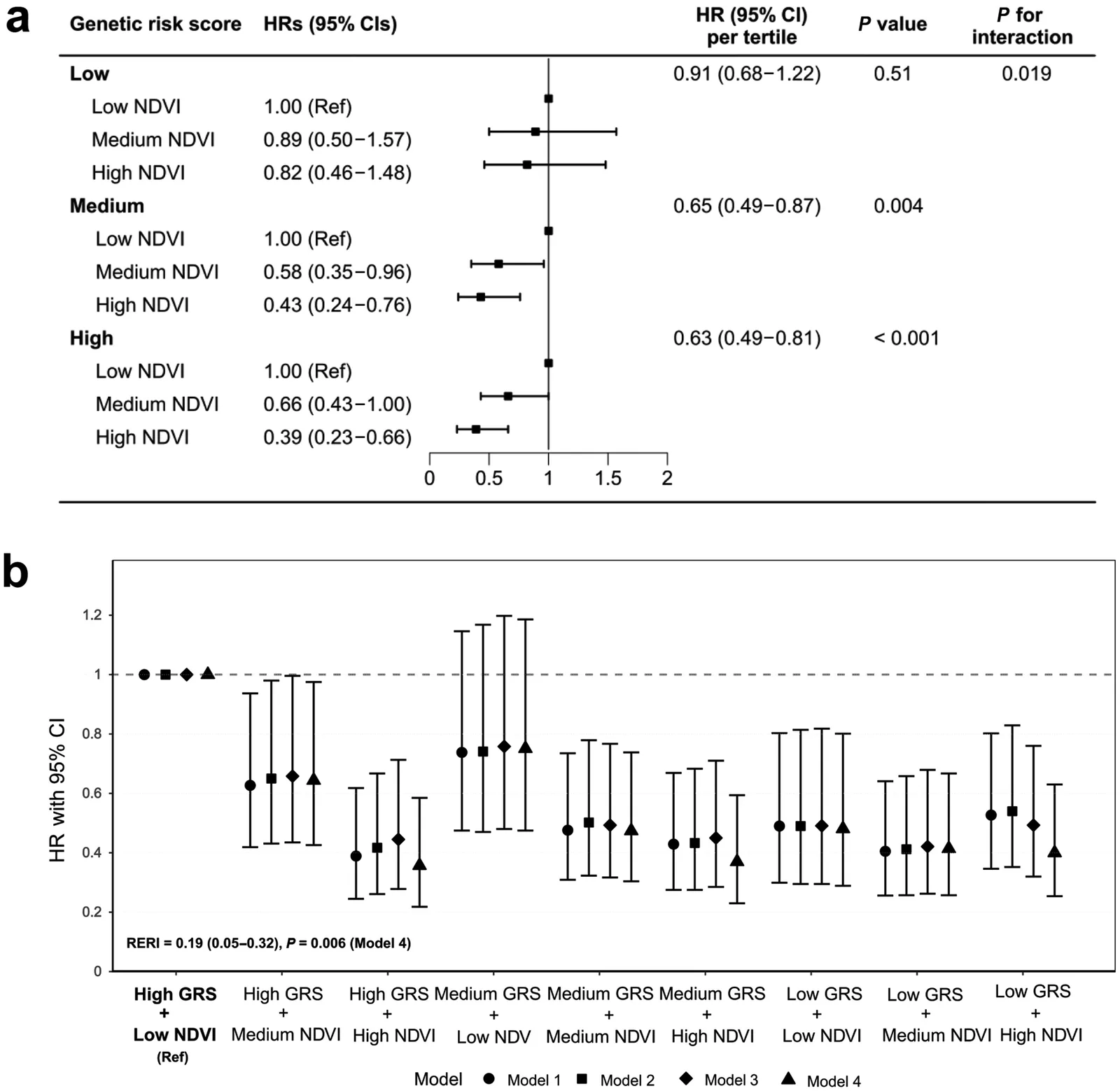
Joint effects of T2D-specific GRS and residential greenness on incident T2D. (a) Associations between residential greenness, assessed by satellite-derived NDVI, and incident T2D across low, medium, and high GRS groups in the fully adjusted model (Model 4). Low NDVI was used as the reference within each genetic risk stratum. HRs and 95% CIs per tertile increase in NDVI and *P-*value for interaction are shown. (b) Joint associations of GRS and residential greenness with incident T2D across sequentially adjusted models, using participants with high GRS and low NDVI as the reference group. Model 1: adjusted for age and sex. Model 2: Model 1 + education level, smoking status, alcohol drinking, physical activity, and healthy diet score. Model 3: Model 2 + body mass index, systolic blood pressure, total cholesterol, triglyceride, and high-density lipoprotein cholesterol. Model 4: Model 3 + time-varying PM_2.5_.

After stratification by genetic risk, higher residential greenness was associated with a lower risk of incident T2D among indivi­duals with medium and high genetic risk (Fig. 3a; Supplementary Figs. S1a and S2a, Model 4). Compared with participants living in areas with the lowest greenness, those residing in areas with the highest residential greenness level had a 57% and 61% lower risk of T2D in the medium and high genetic risk groups, respectively. A significant inverse dose-response association was observed between residential greenness and incident T2D among participants with medium and high genetic risk. Specifically, each tertile increase in NDVI was associated with a 35% reduction in T2D risk among individuals in the medium genetic risk group (HR per tertile = 0.65, 95% CI: 0.49–0.87, *P *= 0.004) and a 37% reduction among those in the high genetic risk group (HR per tertile = 0.63, 95% CI: 0.49–0.81, *P *< 0.001). In contrast, no significant trend was observed among participants with low genetic risk (HR per tertile = 0.91, 95% CI: 0.68–1.22, *P *= 0.51).

A statistically significant multiplicative interaction between residential greenness and GRS was detected (*P* for interaction = 0.019), indicating that the inverse association between greenness exposure and T2D risk was modified by genetic susceptibility (Fig. 3a, Model 4).

Fig. 3b shows the joint association between residential greenness and genetic risk with incident T2D, with participants in the high genetic risk and low residential greenness group serving as the reference. Compared with this group, the combination of high genetic risk and the highest level of residential greenness was associated with a lower risk of incident T2D than the combination of low genetic risk and the lowest level of residential greenness. Among participants with low genetic risk, HRs were similar across the three residential greenness levels.

Consistent with these patterns, we observed a significant additive interaction between residential greenness and genetic risk (RERI = 0.19, 95% CI: 0.05–0.32, *P *= 0.006). The positive RERI indicated that the absolute risk reduction associated with higher resi­dential greenness was greater among individuals with medium and high genetic risk than among those with low genetic risk (Fig. 3b, Model 4). The sensitivity analysis revealed that the additive interaction between residential greenness and genetic risk remained significant, even when NDVI was measured using different buffer ranges (Supplementary Figs. S1b and S2b).

## Discussion

In this prospective cohort study among Chinese adults, higher resi­dential greenness was independently associated with a reduced risk of T2D and more favorable glucose homeostasis, independent of major confounders. Importantly, these associations were modified by genetic susceptibility, with evidence of significant gene-environment interactions on both the multiplicative and additive scales. Participants exposed to both low greenness and high genetic risk exhibited the highest T2D risk, whereas greater greenness exposure was associated with a graded attenuation of risk among those with moderate to high genetic predisposition. These findings highlight the potential role of residential greenness as a modifiable environmental factor for diabetes prevention, particularly among individuals with high genetic predisposition.

Findings from previous studies on residential greenness and T2D risk have been inconsistent [4, 6–8, 16]. A Danish study of 1.9 million adults found that the absence of green space within 150 m and 1000 m of residences is associated with an increased risk of T2D [7]. Similarly, a recent Chinese cohort reported that each interquartile range increase in cumulative average NDVI within the 250-m buffer was associated with a 44% reduction in diabetes risk (HR = 0.56, 95% CI: 0.51–0.61) [6]. In contrast, other studies, such as the KORA cohort, found weaker or null associations [4], likely due to differences in urban structure, greenness measures, follow-up duration, or methods for outcome ascertainment. In the present study, higher residential greenness was consistently associated with a lower risk of T2D, and this association remained robust across sensitivity and stratified analyses. Importantly, diabetes diagnosis was based on standardized assessments of fasting and post-load glucose and glycated hemoglobin A1c (HbA1c), rather than self-reported or record-based data, providing more objective evidence for a protective role of greenness in diabetes prevention.

Although the biological mechanisms underlying the protective effect of greenness on T2D risk are not fully understood, several mechanisms have been proposed, including increased physical activity, healthier body weight, and reduced exposure to air pollution [17, 18]. A systematic review of 19 studies reported that access to neighborhood green spaces promotes physical activity and is associated with lower levels of overweight and obesity, thereby potentially reducing the risk of T2D [18]. In our analyses, adjustment for physical activity and BMI had little impact on the inverse association between residential greenness and T2D risk, whereas further adjustment for time-varying PM_2.5_ strengthened this association. These findings suggest that air pollution may play a role in shaping the observed association between residential greenness and T2D risk. This interpretation aligns with findings from Cui *et al.* [17] and Sun *et al.* [19], which showed that green space exposure may mitigate the associations of PM_2.5_ and its components with T2D risk. Green environments may improve insulin sensitivity and β-cell function, potentially via attenuation of chronic inflammation and lipid dysregulation induced by air pollution. Other unmeasured pathways, including stress reduction and modulation of hypothalamic-pituitary-adrenal axis activity, as well as improved sleep and reduced noise exposure, may also contribute, although these were not measured in our study [20].

The observed associations between residential greenness and HOMA indices (HOMA-IR and HOMA-B), which are well-established markers of T2D risk [21], may further support these potential mechanisms. In our prospective analyses, residential greenness was inversely associated with HOMA-IR and positively associated with HOMA-B, even after adjustment for PM_2.5_, suggesting that improvements in insulin sensitivity and β-cell function may partially mediate the protective effect of greenness on T2D risk. These results are consistent with the findings from cross-sectional studies in both Chinese [9] and German populations [22], which reported similar associations between higher residential greenness and more favorable insulin resistance and secretion profiles, although some estimates were attenuated after adjusting for air pollutants.

Beyond established metabolic mediators, individual genetic susceptibility to T2D may further shape the health benefits of greenness. In the current study, we observed both multiplicative and additive interactions between residential greenness and genetic susceptibility in relation to incident T2D. On the multiplicative scale, higher levels of greenness were associated with a progressively lower risk of T2D among participants with medium and high genetic risk, whereas little variation in risk was observed across greenness levels among those with low genetic risk. These findings indicate that genetic susceptibility modifies the relative association between greenness exposure and T2D risk. Notably, this is inconsistent with results from the UK Biobank [14], which identified only a multiplicative interaction, suggesting that greenness conferred greater benefits among low-risk individuals. Differences in study design and population cha­racteristics may account for this discrepancy, as the UK Biobank analysis was cross-sectional and relied on self-reported diabetes status, whereas our prospective cohort incorporated clinically verified diagnoses based on oral glucose tolerance tests (OGTT) and HbA1c, minimizing potential reverse causation and misclassification. Importantly, a positive additive interaction (RERI = 0.19) indicates that the absolute protective effect of residential greenness on T2D risk was greater among individuals with high genetic susceptibility than expected based on the sum of their individual effects. From a public health perspective, this pattern implies that increases in residential greenness may yield disproportionately larger absolute risk reductions in populations at elevated genetic risk, thereby supporting the potential role of environmental interventions in mitigating genetically driven T2D risk. Notably, several single-nucleotide polymorphisms (SNPs) in our T2D-specific GRS (e.g., TCF7L2, KCNQ1, SLC30A8, FTO, and MTNR1B) are involved in pathways related to inflammation, β-cell function, energy meta­bolism, and circadian regulation [23], providing plausible biological mechanisms underlying the observed gene-environment interplay.

Our study has several notable strengths. To our knowledge, this is the first prospective cohort study to comprehensively evaluate gene-environment interactions between T2D-specific genetic risk and residential greenness exposure, using objective diabetes diagnoses based on fasting and postprandial glucose as well as HbA1c levels. Second, the availability of fasting glucose and insulin measurements at follow-up enabled the calculation of HOMA-IR and HOMA-B, key indicators of glucose homeostasis. Third, residential greenness was precisely quantified using the NDVI derived from MODIS satellite data, which was spatially matched to participants’ residential addresses, providing an objective, high-resolution, and standardized measure of individual green space exposure. Fourth, detailed information on lifestyle factors, metabolic profiles, and long-term air pollution exposure allowed rigorous adjustment for potential confounders, thereby strengthening the validity of our findings.

In conclusion, residential greenness is inversely associated with incident T2D, and genetic susceptibility significantly modified these associations, with the greatest protective effects observed among those at medium and high genetic risk. These findings underscore the importance of considering gene-environment interactions and support the role of environmental interventions as population-wide strategies, with potential for enhanced impact among genetically susceptible subgroups, in reducing the burden of T2D.

### Limitations of the study

Our study has several limitations that merit attention. First, the mean follow-up period was relatively short (3.8 years), which may limit the ability to capture long-term effects. Second, although NDVI exposure was updated annually during follow-up to reflect temporal changes in vegetation, these estimates were linked to participants’ baseline residential address. We were unable to reconstruct participants’ address histories, and lacked information on greenness encountered outside the home (e.g., workplace or other routinely visited locations) [24]. Together, these constraints may introduce exposure misclassification by failing to incorporate individuals’ spatiotemporal activity patterns, potentially affecting the magnitude of the estimated associations [25]. Third, NDVI reflects overall vegetation but does not capture green space quality, accessibility, or differences between structured and unstructured environments. Fourth, this study was conducted in a Chinese population from a single county in Zhejiang Province, which may limit the generalizability of our findings to other ethnic groups and to other regions of China, particularly given potential rural–urban heterogeneity. Finally, because T2D diagnoses were primarily captured at scheduled follow-up visits, some partici­pants may have developed diabetes before the recorded visit date, leading to potential misclassification of incident timing; this limitation may bias HR estimates toward the null.

## Materials and methods

### Study population

In this study, we included 9902 participants aged 40 years or older, recruited between March 2011 and August 2011 in Jiashan County, Zhejiang Province, China [26, 27]. The county is located in the northeastern part of the province (approximately 30.75°N, 120.75°E). It covers 506.87 km^2^ and had a population of 385 278 in 2011, with 54.18% living in urban areas. From 2014 to 2016, partici­pants were invited to attend an in-person follow-up assessment. Trained personnel collected information on lifestyle risk factors and medical history using the same standardized questionnaire administered at baseline. Anthropometric measurements, OGTT, and blood samples were obtained following the same protocol as that used in the baseline examination. For the present analysis, we excluded participants with diabetes at baseline (*n *= 1927), those with missing data on incident T2D during follow-up (*n *= 83), and those with missing data on residential greenness (*n *= 31), leaving 7861 individuals for the final analysis (Supplementary Fig. S3).

### Residential greenness exposure assessment

NDVI data were obtained from the MODIS within the National Aeronautics and Space Administration (NASA) Earth Observing System. MODIS vegetation indices at the global scale are designed to allow consistent assessment of vegetation dyna­mics across different regions and time periods. The daily indices are derived from reflectance in the blue, red, and near-infrared wavelengths, centered at 469, 645, and 858 nm, respectively. The MODIS NDVI complements NOAA’s AVHRR NDVI datasets, providing continuity for long-term temporal analyses based on this extensive historical record [28]. NDVI is a standardized spectral indicator calculated from the contrast between red light absorption and near-infrared reflectance relative to the total incoming visible and near-infrared radiation, and is widely applied to assess vegetation vigor, coverage, and biomass based on multispectral remote-sensing data [12]. The global MODIS NDVI Version 5 dataset (NASA, Washington, D.C., USA) is gene­rated at 16-day intervals with a spatial resolution of 250 m, yielding index values ranging from −1.0 to 1.0. Values approaching −1.0 typically represent open water, whereas values near zero correspond to barren surfaces or snow cover. Increasing positive values reflect progressively denser vegetation, with indices of approximately 0.2–0.4 indicating grasslands or shrublands and values approaching 1.0 characteristic of dense tropical forests [5, 6]. Consequently, NDVI serves as an integrated proxy for overall environmental greenness. The MODIS Vegetation Indices (MOD13) product suite has achieved validation stage 3, with NDVI accuracy under ideal conditions (clear skies, low aerosol loading, and sensor view angle < 30°) within ± 0.025. When pixel quality is uncertain, the error may increase to approximately 0.04–0.1 [29]. Previous validation studies have demonstrated strong correlations between NDVI-derived estimates and ground-based measurements of vegetative cover, supporting its utility in epidemiological research [5, 8].

In this study, the surrounding greenness for each participant was quantified using the satellite-derived NDVI. The values range from 0 to 1.0, with higher values reflecting denser vegetative presence. Residential greenness was estimated by linking NDVI imagery to the longitude and latitude coordinates of each residential address and calculating mean greenness levels within a 500-m radial buffer, which is a commonly used indicator of local green space exposure [30]. To assess the robustness of the findings to buffer selection, we conducted sensitivity analyses using the mean NDVI within 250-m and 1250-m buffers [31]. To reflect temporal changes in greenness, NDVI was updated annually: for each participant, mean NDVI values were extracted for each year from 2011 to 2016, representing long-term, continuous, time-varying greenness exposure over follow-up. The spatial distribution of residential greenness in Jiashan County is shown in Fig. 1.

### Ascertainment of T2D and measurement of glucose homeostasis markers

T2D at baseline and incident T2D during follow-up were ascertained based on any of the following: (i) fasting plasma glucose ≥ 7.0 mmol/L; (ii) 2-h post-OGTT plasma glucose ≥ 11.1 mmol/L; (iii) HbA1c ≥ 6.5%; or (iv) a self-reported physician diagnosis of diabetes. Insulin resistance was quantified using HOMA-IR, and pancreatic β-cell function was assessed using HOMA-B at follow-up visits.

### GRS

Of the 7861 individuals included in the final analysis, 5389 participants provided DNA samples and were included in the GRS analyses. The procedures for genotyping, quality control, and genotype imputation have been detailed elsewhere [32, 33]. We performed whole-genome imputation using the 1000 Genomes Project (Phase 3) East Asian reference panel. We generated a T2D-specific GRS based on 89 SNPs that met stringent quality control criteria (including an imputation Info score > 0.7, a genotype call rate > 98%, and a minor allele frequency > 0.01). These SNPs were identified by matching our QC-passed variants with 183 loci recently reported as genome-wide significant for T2D in East Asian populations [34]. Detailed information on the selected SNPs is provided in Supplementary Table S6. The GRS was derived using a standard weighted approach according to the following formula:

GRS = (β_1_ × SNP_1_ + β_2_ × SNP_2_ + … + β_89_ × SNP_89_) × (89/sum of the β coefficients)

where each SNP corresponds to the count of diabetes risk alleles (0, 1, 2), and β represents the effect estimate for the association between the variant and T2D. In the present analysis, diabetes GRS values ranged from 4.33 to 7.68, with higher scores reflecting greater inherited susceptibility to the disease.

### Covariates

Data on covariates, including age, sex, education level, smoking status, habitual alcohol drinking, physical activity, and diet, were collected at baseline using standard questionnaires. Physical activity was quantified using the short form of the International Physical Activity Questionnaire and dietary intake during the previous year was assessed with a validated dietary questionnaire. A diet quality score was then derived according to American Heart Association guidelines. This score has been shown to predict health outcomes in Chinese populations [35]. BMI was computed as weight divided by height squared. Blood pressure was recorded by trained nurses according to standardized protocols. Blood samples were collected after overnight fasting, and lipid concentrations were determined using an auto analyzer (Abbott Laboratories, IL).

Air pollution exposure was assessed as the annual mean concentration of PM_2.5_ from 2011 to 2016 at a spatial resolution of 1 km^2^. Daily PM_2.5_ levels were then estimated from satellite-derived data through space-time extremely randomized tree (STET) models, available via the ChinaHighPM_2.5_ dataset. Daily PM_2.5_ concentrations were derived from hourly observations provided by the China National Environmental Monitoring Center (CNEMC). However, the hourly data were not directly used as an exposure metric in the analysis [36]. The STET approach integrates satellite aerosol optical depth (AOD) with meteorological, topographic, land-use, and emission-related predictors to produce high-resolution exposure surfaces. In the source study, model performance was assessed using both out-of-sample and out-of-station cross-validation, yielding *R*^2^ values of 0.89 and 0.88, respectively, with root-mean-square error of 10.33–10.93 µg/m^3^ and mean absolute error of 6.69–7.15 µg/m^3^ (mean relative error: 21.28%–23.69%). These validation results indicate good predictive accuracy and spatial generalizability for epidemiologic exposure assessment [37].

### Statistical analysis

Continuous variables were summarized as mean (SD), and categorical variables as number (percentage). Because both NDVI and PM_2.5_ vary annually, we applied a time-varying Cox proportional hazards model to estimate the association between residential greenness exposure and incident T2D. Schoenfeld residual tests indicated violation of the proportional hazards assumption in a standard Cox model, supporting the use of a time-varying Cox approach. Person-years were calculated from baseline until the date of diabetes diagnosis, or the end of follow-up, whichever came first. HRs and 95% CIs were estimated for greenness treated as both a continuous variable and in tertiles. Covariates included baseline age, sex, education level, smoking status, drinking habits, physical activity, diet score, BMI, systolic blood pressure, total cholesterol, triglyceride, high-density lipoprotein chole­sterol, and time-varying PM_2.5_. The *P* for trend value was evaluated by modeling the medium greenness value within each tertile as a continuous variable.

To systematically control for potential confounding factors, progressively adjusted models were constructed. Model 1 was adjusted for age and sex. Model 2 was further adjusted for education level, smoking status, habitual alcohol drinking, physical activity, and healthy diet score. Model 3 additionally included BMI, systolic blood pressure, total cholesterol, triglyceride, and high-density lipoprotein cholesterol. Model 4 further incorporated time-varying PM_2.5_ exposure to account for temporal variation in air pollution. The *P* for trend was evaluated by modeling the medium greenness value within each tertile as a continuous variable.

Potential nonlinear associations between greenness and diabetes risk, as well as glucose homeostasis markers, were examined using time-varying Cox regression and linear regression models with RCS of NDVI, with 0.2 specified as the reference value. Knots were placed at the 10th, 50th, and 90th percentiles of the NDVI distribution.

We further conducted stratified analyses by sex (male and female), age (< 55 and ≥ 55 years), smoking status (current smoker or not), drinking status (current drinker or not), physical activity (high or low), BMI (< 28 kg/m^2^ and ≥ 28 kg/m^2^), and GRS (high, medium, and low). Subgroup differences were evaluated by including multiplicative interaction terms between greenness and each covariate.

To examine the joint effects of residential greenness and genetic susceptibility, participants were categorized into nine groups based on greenness tertiles and GRS levels (low, medium, and high). Outcome estimates for each group were compared with the reference group characterized by low greenness and high GRS. Furthermore, RERI and 95% CI were calculated [38].

In the sensitivity analyses, we used NDVI estimated within 250-m and 1250-m buffers to assess the robustness of the findings, with the same covariate adjustment and modeling specifications.

R software, version 4.4.1, was used for the analyses. Two-sided statistical tests were conducted.

## Supplementary Material

loag019_Supplementary_Data

## Data Availability

The data are available from the corresponding authors upon reasonable request.
