# Supplementary material for: Joint effects of residential greenness and genetic susceptibility on type 2 diabetes: a prospective cohort study using satellite-derived Normalized Difference Vegetation Index
Source: Life Metab. 2026 Jul 8;5(5):loag019. doi: 10.1093/lifemeta/loag019 (PMC13425641; doi:10.1093/lifemeta/loag019)
Supplement: loag019_Supplementary_Data [file loag019_supplementary_data.docx]

**Joint effects of residential greenness and genetic susceptibility on type 2 diabetes: a prospective cohort study using satellite-derived NDVI**

Yonghua Yu^1,2,‡^, Junru Fan^1,2,‡^, Qiuyu Cao^1,2,‡^, Dong Li^3,‡^, Hong Lin^1,2,‡^, Zhen Ye^4^, Ruizhi Zheng^1,2^, Yu Xu^1,2^, Min Xu^1,2^, Mian Li^1,2^, Libin Zhou^1,2^, Shuangyuan Wang^1,2^, Tiange Wang^1,2^, Zhiyun Zhao^1,2^, Jie Zheng^1,2^, Guang Ning^1,2^, Weiqing Wang^1,2^, Ruying Hu^4,^^*^, Yufang Bi^1,2,*^, and Jieli Lu^1,2,*^

^1^Department of Endocrine and Metabolic Diseases, Shanghai Institute of Endocrine and Metabolic Diseases, Ruijin Hospital, Shanghai Jiao Tong University School of Medicine, Shanghai 200025, China

^2^Shanghai National Clinical Research Center for Endocrine and Metabolic Diseases, Key Laboratory for Endocrine and Metabolic Diseases of the National Health Commission of the PR China, Shanghai National Center for Translational Medicine, Shanghai Digital Medicine Innovation Center, Ruijin Hospital, Shanghai Jiao-Tong University School of Medicine, Shanghai 200025, China

^3^Institute for Urban Governance and Sustainable Development, Tsinghua University, Beijing 100084, China

^4^Zhejiang Provincial Center for Disease Control and Prevention, Hangzhou, Zhejiang 310051, China

^‡^These authors contributed equally to this work.

^*^**Corresponding authors.** National Clinical Research Center for Metabolic Diseases, Rui Jin Hospital, Shanghai Jiao Tong University School of Medicine, 197 Rui Jin 2nd Road, Shanghai 200025, China. E-mail: ljl11319@rjh.com.cn (J.L.); National Clinical Research Center for Metabolic Diseases, Rui Jin Hospital, Shanghai Jiao Tong University School of Medicine, 197 Rui Jin 2nd Road, Shanghai 200025, China. E-mail: byf10784@rjh.com.cn (Y.B.); Zhejiang Provincial Center for Disease Control and Prevention, 3399 Binsheng Road, Hangzhou 310051, China. E-mail: ryhu@cdc.zj.cn (R.H.)

**Supplementary Material**

**Supplementary Figure S1** Joint effects of type 2 diabetes-specific genetic risk score and residential greenness (250 m) on incident T2D. Model 1: adjusted for age and sex. Model 2: model 1 + education level, smoking status, alcohol drinking, physical activity, and healthy diet score. Model 3: model 2 + body mass index, systolic blood pressure, total cholesterol, triglyceride, high-density lipoprotein cholesterol. Model 4: model 3 + time-varying PM_2.5_.

**
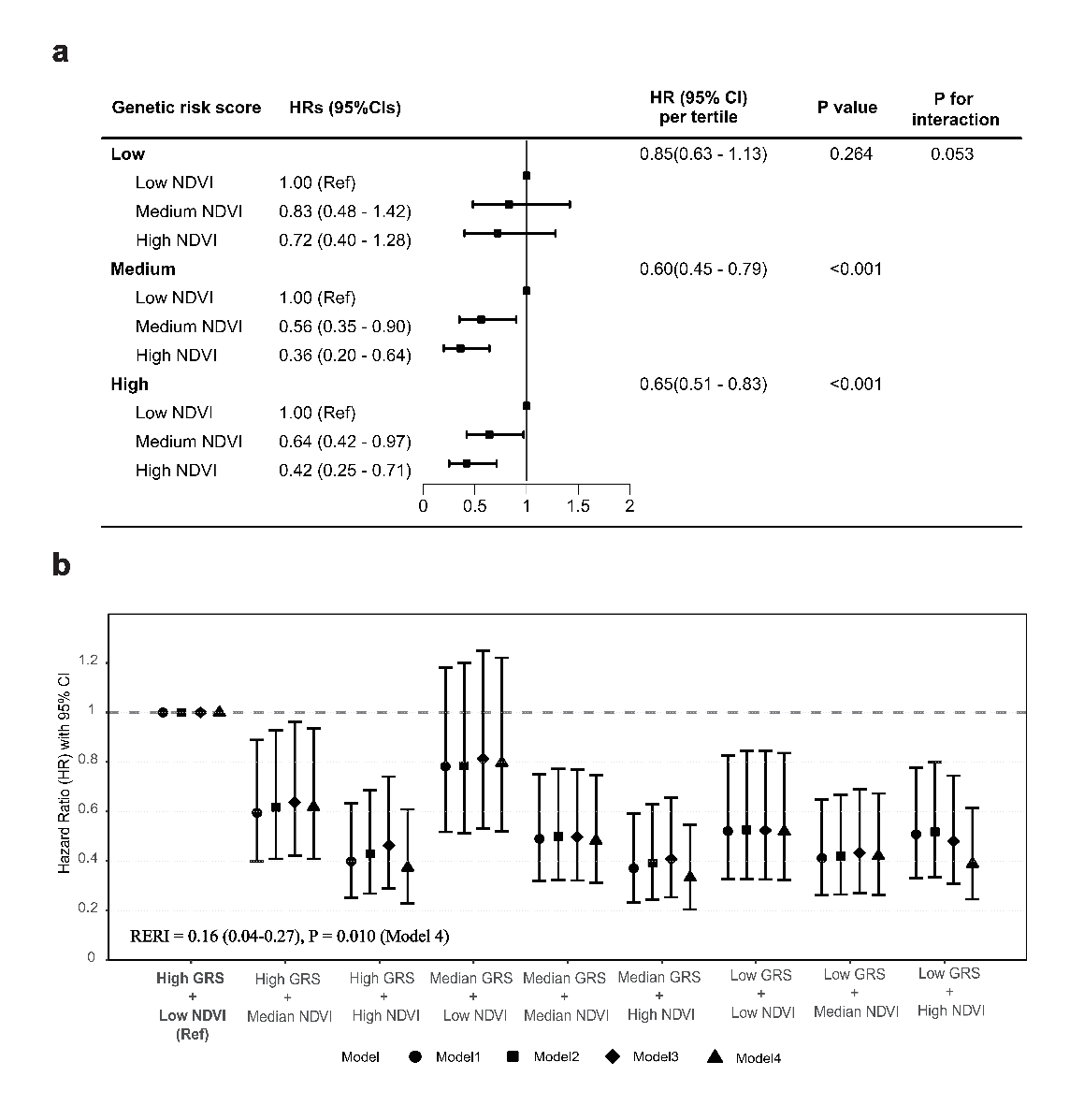
**

**Supplementary Figure S2** Joint effects of type 2 diabetes-specific genetic risk score and residential greenness (1250 m) on incident T2D. Model 1: adjusted for age and sex. Model 2: model 1 + education level, smoking status, alcohol drinking, physical activity, and healthy diet score. Model 3: model 2 + body mass index, systolic blood pressure, total cholesterol, triglyceride, high-density lipoprotein cholesterol. Model 4: model 3 + time-varying PM_2.5_.

**
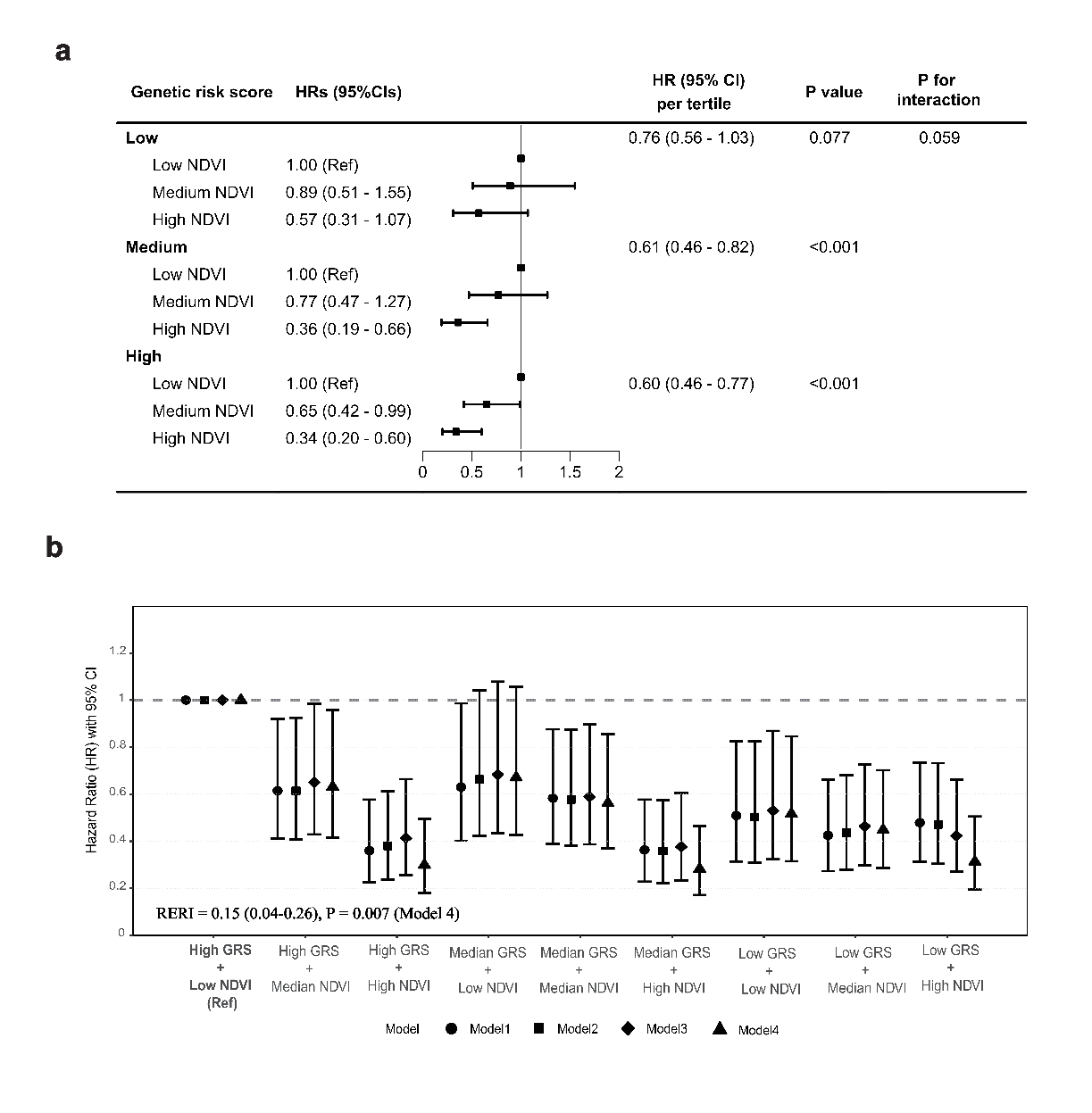
**

**Supplementary Figure S3** Flow chart.

**
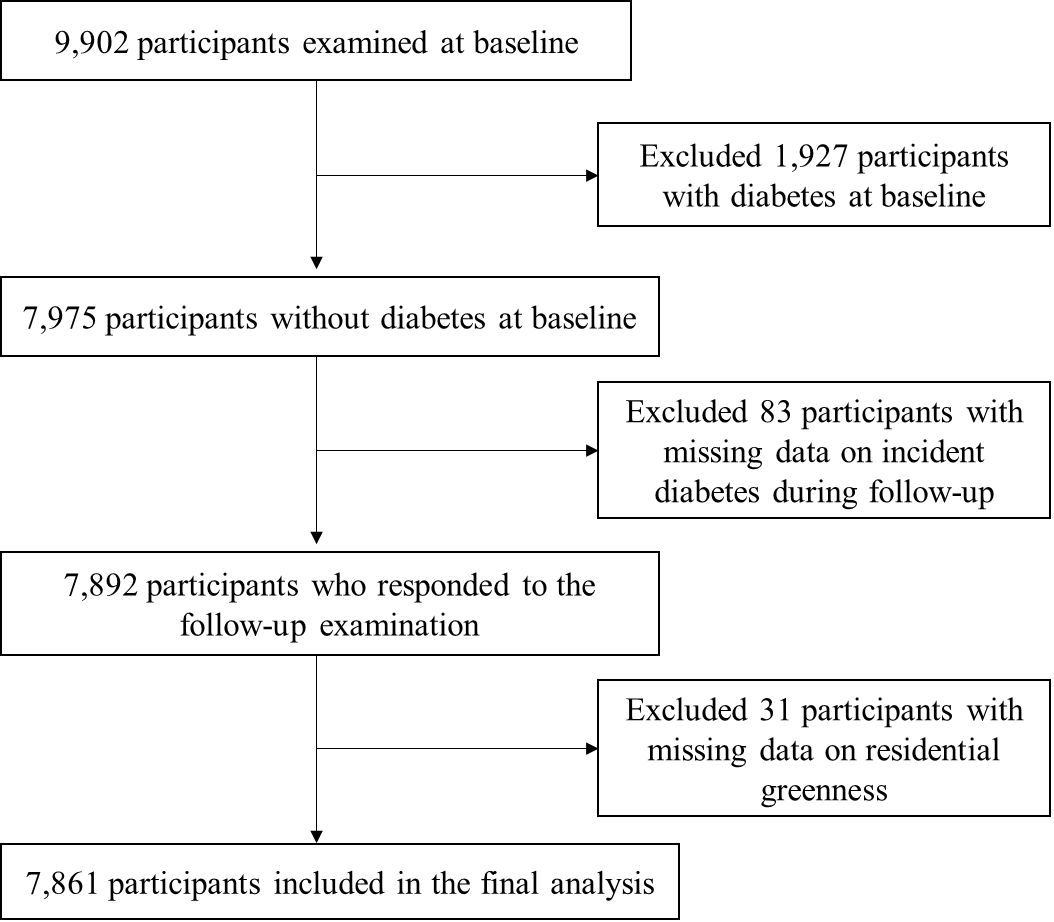
**

**Supplementary Table S1** Association between residential greenness (250 m) and incident T2D.

| Model | HR (95% CI) for 0.1-unit increase in residential greenness | HR (95% CI) in tertiles of residential greenness | | | *P* for trend |
| --- | --- | --- | --- | --- | --- |
|  |  | Tertile 1 | Tertile 2 | Tertile 3 |  |
| Model 1 | 0.68 (0.59-0.78) | 1.00 (Ref) | 0.70 (0.57–0.86) | 0.53 (0.42–0.66) | < 0.0001 |
| Model 2 | 0.68 (0.59-0.79) | 1.00 (Ref) | 0.70 (0.57–0.87) | 0.54 (0.43–0.68) | < 0.0001 |
| Model 3 | 0.68 (0.59-0.78) | 1.00 (Ref) | 0.71 (0.58–0.88) | 0.55 (0.44–0.69) | < 0.0001 |
| Model 4 | 0.57 (0.49-0.67) | 1.00 (Ref) | 0.71 (0.57–0.88) | 0.46 (0.36–0.59) | < 0.0001 |

Model 1: adjusted for age and sex. Model 2: model 1 + education level, smoking status, alcohol drinking, physical activity, and healthy diet score. Model 3: model 2 + body mass index, systolic blood pressure, total cholesterol, triglyceride, and high-density lipoprotein cholesterol. Model 4: model 3 + time-varying PM_2.5_.

**Supplementary Table S2** Association between residential greenness (1250 m) and incident T2D.

| Model | HR (95% CI) for 0.1-unit increase in residential greenness | HR (95% CI) in tertiles of residential greenness | | | *P* for trend |
| --- | --- | --- | --- | --- | --- |
|  |  | Tertile 1 | Tertile 2 | Tertile 3 |  |
| Model 1 | 0.56 (0.46-0.67) | 1.00 (Ref) | 0.74 (0.60–0.91) | 0.50 (0.40–0.63) | < 0.0001 |
| Model 2 | 0.56 (0.47-0.68) | 1.00 (Ref) | 0.74 (0.60–0.91) | 0.49 (0.39–0.63) | < 0.0001 |
| Model 3 | 0.56 (0.47-0.68) | 1.00 (Ref) | 0.76 (0.62–0.95) | 0.49 (0.39–0.63) | < 0.0001 |
| Model 4 | 0.41 (0.33-0.50) | 1.00 (Ref) | 0.76 (0.62–0.95) | 0.37 (0.29–0.49) | < 0.0001 |

Model 1: adjusted for age and sex. Model 2: model 1 + education level, smoking status, alcohol drinking, physical activity, and healthy diet score. Model 3: model 2 + body mass index, systolic blood pressure, total cholesterol, triglyceride, high-density lipoprotein cholesterol. Model 4: model 3 + time-varying PM_2.5_.

**Supplementary Table S3** Stratified analysis on associations of greenness (250 m) with incident T2D.

| Subgroups | HR (95% CI) in tertiles of residential greenness | | | *P* for trend | *P* for interaction |
| --- | --- | --- | --- | --- | --- |
|  | Tertile 1 | Tertile 2 | Tertile 3 |  |  |
| Sex |  |  |  |  | 0.787 |
| Male | 1.00 (Ref) | 0.61 (0.45–0.83) | 0.48 (0.34 - 0.67) | < 0.001 |  |
| Female | 1.00 (Ref) | 0.82 (0.61–1.11) | 0.43 (0.30 - 0.63) | < 0.001 |  |
| Age |  |  |  |  | 0.889 |
| < 55 years | 1.00 (Ref) | 0.69 (0.52–0.93) | 0.51 (0.37–0.71) | < 0.001 |  |
| ≥ 55 years | 1.00 (Ref) | 0.74 (0.54–1.01) | 0.40 (0.27–0.59) | < 0.001 |  |
| Current smoking |  |  |  |  | 0.861 |
| No | 1.00 (Ref) | 0.71 (0.55–0.91) | 0.47 (0.35–0.64) | < 0.001 |  |
| Yes | 1.00 (Ref) | 0.72 (0.50–1.05) | 0.43 (0.28–0.67) | < 0.001 |  |
| Current drinking |  |  |  |  | 0.446 |
| No | 1.00 (Ref) | 0.75 (0.58–0.96) | 0.50 (0.37–0.67) | < 0.001 |  |
| Yes | 1.00 (Ref) | 0.60 (0.40–0.90) | 0.35(0.21–0.58) | < 0.001 |  |
| Physical activity |  |  |  |  | 0.589 |
| Inactive | 1.00 (Ref) | 0.71 (0.57–0.90) | 0.47 (0.36–0.61) | < 0.001 |  |
| Active | 1.00 (Ref) | 0.76 (0.41–1.38) | 0.44 (0.19–1.00) | 0.049 |  |
| Body mass index |  |  |  |  | 0.425 |
| < 28 kg/m2 | 1.00 (Ref) | 0.72 (0.57–0.90) | 0.46 (0.35–0.60) | < 0.001 |  |
| ≥ 28 kg/m2 | 1.00 (Ref) | 0.68 (0.39–1.18) | 0.43 (0.23–0.80) | 0.007 |  |

Models are adjusted for age, sex, education level, smoking status, alcohol drinking, physical activity, healthy diet score, body mass index, systolic blood pressure, total cholesterol, triglyceride, high-density lipoprotein cholesterol, and time-varying PM_2.5_.

**Supplementary Table S4** Stratified analysis on associations of greenness (1250 m) with incident T2D.

| Subgroups | HR (95% CI) in tertiles of residential greenness | | | *P* for trend | *P* for interaction |
| --- | --- | --- | --- | --- | --- |
|  | Tertile 1 | Tertile 2 | Tertile 3 |  |  |
| Sex |  |  |  |  | 0.200 |
| Male | 1.00 (Ref) | 0.79 (0.59–1.07) | 0.44 (0.30–0.63) | < 0.001 |  |
| Female | 1.00 (Ref) | 0.75 (0.55–1.02) | 0.31 (0.21–0.47) | < 0.001 |  |
| Age |  |  |  |  | 0.682 |
| < 55 years | 1.00 (Ref) | 0.76 (0.57–1.02) | 0.45 (0.32–0.64) | < 0.001 |  |
| ≥ 55 years | 1.00 (Ref) | 0.76 (0.55–1.04) | 0.29 (0.19–0.44) | < 0.001 |  |
| Current smoking |  |  |  |  | 0.933 |
| No | 1.00 (Ref) | 0.77 (0.59–1.00) | 0.38 (0.28–0.53) | < 0.001 |  |
| Yes | 1.00 (Ref) | 0.76 (0.52–1.10) | 0.36 (0.23–0.58) | < 0.001 |  |
| Current drinking |  |  |  |  | 0.954 |
| No | 1.00 (Ref) | 0.75 (0.58–0.96) | 0.40 (0.29–0.54) | < 0.001 |  |
| Yes | 1.00 (Ref) | 0.81 (0.54–1.22) | 0.30 (0.17–0.52) | < 0.001 |  |
| Physical activity |  |  |  |  | 0.933 |
| Inactive | 1.00 (Ref) | 0.72 (0.57–0.91) | 0.38 (0.29–0.51) | < 0.001 |  |
| Active | 1.00 (Ref) | 1.14 (0.60–2.16) | 0.39 (0.15–0.97) | 0.055 |  |
| Body mass index |  |  |  |  | 0.584 |
| < 28 kg/m2 | 1.00 (Ref) | 0.75 (0.59–0.94) | 0.37 (0.27–0.49) | < 0.001 |  |
| ≥ 28 kg/m2 | 1.00 (Ref) | 0.81 (0.47–1.41) | 0.38 (0.19–0.75) | 0.006 |  |

Models are adjusted for age, sex, education level, smoking status, alcohol drinking, physical activity, healthy diet score, body mass index, systolic blood pressure, total cholesterol, triglyceride, high-density lipoprotein cholesterol, and time-varying PM_2.5_.

**Supplementary Table S5** Association between genetic risk score and incident type 2 diabetes.

| Model | HR (95% CI) in tertiles of GRS | | | *P* for trend |
| --- | --- | --- | --- | --- |
|  | Tertile 1 | Tertile 2 | Tertile 3 |  |
| Model 1 | 1.00 (Ref) | 1.10 (0.83–1.46) | 1.34 (1.03–1.76) | 0.030 |
| Model 2 | 1.00 (Ref) | 1.10 (0.83–1.47) | 1.36 (1.04–1.80) | 0.025 |
| Model 3 | 1.00 (Ref) | 1.15 (0.86–1.54) | 1.44 (1.09–1.91) | 0.009 |

Model 1: adjusted for age and sex. Model 2: model 1 + education level, smoking status, alcohol drinking, physical activity, and healthy diet score. Model 3: model 2 + body mass index, systolic blood pressure, total cholesterol, triglyceride, high-density lipoprotein cholesterol.

**Supplementary Table S6** Summary characteristics of 90 SNPs associated with type 2 diabetes.

| No. | SNP | Effect allele | Non-effect allele | β coefficient | Nearest gene |
| --- | --- | --- | --- | --- | --- |
| 1 | rs11205766 | T | A | 0.086178 | *FAF1* |
| 2 | rs2269245 | G | A | 0.058269 | *PGM1* |
| 3 | rs532504 | A | G | 0.058269 | *LINC01741; SEC16B* |
| 4 | rs1327123 | C | G | 0.039221 | *COLGALT2; TSEN15* |
| 5 | rs1260326 | C | T | 0.067659 | *GCKR* |
| 6 | rs75536691 | A | G | 0.182322 | *GRB14* |
| 7 | rs75179644 | T | C | 0.076961 | *LINC01878; MIR4776-2* |
| 8 | rs11926494 | G | A | 0.113329 | *UBE2E2* |
| 9 | rs9859381 | G | T | 0.039221 | *CASR* |
| 10 | rs1850421 | A | C | 0.04879 | *MBNL1; P2RY1* |
| 11 | rs13092876 | A | G | 0.122218 | *IGF2BP2* |
| 12 | rs13086331 | T | C | 0.04879 | *LINC01991; LPP-AS2* |
| 13 | rs7656416 | C | T | 0.10436 | *CTBP1-AS2; MAEA* |
| 14 | rs10938398 | A | G | 0.04879 | *GNPDA2; GABRG1* |
| 15 | rs28599782 | A | G | 0.067659 | *MOB1B* |
| 16 | rs16902871 | G | A | 0.058269 | *RANBP3L* |
| 17 | rs74334916 | C | A | 0.067659 | *PARP8* |
| 18 | rs256904 | T | A | 0.076961 | *C5orf67* |
| 19 | rs2126736 | A | G | 0.039221 | *ANKRD31; HMGCR* |
| 20 | rs6556925 | C | A | 0.039221 | *LOC101929710* |
| 21 | rs329122 | A | G | 0.039221 | *JADE2* |
| 22 | rs9379084 | G | A | 0.067659 | *RREB1* |
| 23 | rs9350271 | A | G | 0.19062 | *CDKAL1* |
| 24 | rs76541615 | T | G | 0.076961 | *HCG22* |
| 25 | rs4711389 | A | G | 0.09531 | *SMIM29* |
| 26 | rs4273712 | G | A | 0.04879 | *MIR588; RSPO3* |
| 27 | rs9390022 | T | C | 0.04879 | *LOC153910; HIVEP2* |
| 28 | rs17168486 | T | C | 0.067659 | *DGKB* |
| 29 | rs3735567 | G | A | 0.058269 | *JAZF1* |
| 30 | rs610930 | A | G | 0.067659 | *AUTS2* |
| 31 | rs12698877 | G | A | 0.067659 | *AUTS2* |
| 32 | rs2074120 | A | C | 0.039221 | *CALCR* |
| 33 | rs2233580 | T | C | 0.29267 | *PAX4* |
| 34 | rs1182444 | G | A | 0.04879 | *UBE3C* |
| 35 | rs34642578 | T | C | 0.086178 | *ASAH1* |
| 36 | rs349359 | C | A | 0.039221 | *KCNB2* |
| 37 | rs149265787 | G | A | 0.131028 | *JPH1* |
| 38 | rs896852 | G | T | 0.039221 | *TP53INP1* |
| 39 | rs13266634 | C | T | 0.113329 | *SLC30A8* |
| 40 | rs73708054 | C | T | 0.039221 | *ADCY8; EFR3A* |
| 41 | rs1016565 | A | G | 0.039221 | *DMRT3; LINC01230* |
| 42 | rs4237150 | C | G | 0.067659 | *GLIS3* |
| 43 | rs1328412 | T | C | 0.09531 | *LOC101927450; TLE4* |
| 44 | rs2796441 | G | A | 0.076961 | *LOC101927502* |
| 45 | rs113154802 | C | T | 0.058269 | *PTCH1* |
| 46 | rs529565 | C | T | 0.039221 | *ABO* |
| 47 | rs11257657 | G | C | 0.113329 | *CDC123; CAMK1D* |
| 48 | rs77065181 | A | G | 0.086178 | *PTF1A; C10orf67* |
| 49 | rs1955163 | G | A | 0.04879 | *TSPAN15; NEUROG3* |
| 50 | rs34907385 | C | T | 0.04879 | *LRMDA* |
| 51 | rs1236816 | A | C | 0.039221 | *PTEN* |
| 52 | rs10736116 | C | G | 0.04879 | *ARHGAP19-SLIT1; FRAT1* |
| 53 | rs7895872 | T | G | 0.04879 | *BBIP1* |
| 54 | rs7901695 | C | T | 0.277632 | *TCF7L2* |
| 55 | rs10886863 | C | T | 0.058269 | *WDR11; FGFR2* |
| 56 | rs11043003 | C | T | 0.10436 | *MIR4686; ASCL2* |
| 57 | rs2237897 | C | T | 0.24686 | *KCNQ1* |
| 58 | rs4148646 | C | G | 0.076961 | *ABCC8* |
| 59 | rs4922793 | A | G | 0.039221 | *BDNF* |
| 60 | rs7109575 | G | A | 0.139762 | *ARAP1* |
| 61 | rs10830963 | G | C | 0.039221 | *MTNR1B* |
| 62 | rs80234489 | C | A | 0.10436 | *FAM60A* |
| 63 | rs2583934 | T | G | 0.058269 | *HMGA2* |
| 64 | rs7313668 | T | G | 0.04879 | *PTPRR; TSPAN8* |
| 65 | rs10860209 | C | A | 0.039221 | *NEDD1; RMST* |
| 66 | rs7983505 | T | A | 0.076961 | *LINC00423; KL* |
| 67 | rs123378 | G | A | 0.04879 | *DLEU1* |
| 68 | rs1215468 | A | G | 0.086178 | *LINC01080; SPRY2* |
| 69 | rs9515905 | A | G | 0.076961 | *LINC00379; MIR17HG* |
| 70 | rs12437434 | C | T | 0.04879 | *NYNRIN* |
| 71 | rs61975988 | A | G | 0.039221 | *CLEC14A; LINC00639* |
| 72 | rs8043085 | T | G | 0.04879 | *RASGRP1* |
| 73 | rs8037894 | G | C | 0.076961 | *C2CD4A; C2CD4B* |
| 74 | rs4776970 | A | T | 0.039221 | *MAP2K5* |
| 75 | rs8038760 | A | C | 0.04879 | *SIN3A* |
| 76 | rs952472 | C | A | 0.067659 | *HMG20A* |
| 77 | rs8026714 | A | G | 0.067659 | *PRC1-AS1* |
| 78 | rs117267808 | A | G | 0.10436 | *GP2* |
| 79 | rs1421085 | C | T | 0.131028 | *FTO* |
| 80 | rs12600132 | T | C | 0.039221 | *PKD1L3* |
| 81 | rs6416749 | C | T | 0.04879 | *ZFHX3; HCCAT5* |
| 82 | rs2925979 | T | C | 0.039221 | *CMIP* |
| 83 | rs7502556 | T | C | 0.04879 | *NF1* |
| 84 | rs8064454 | A | C | 0.122218 | *HNF1B* |
| 85 | rs476828 | C | T | 0.086178 | *PMAIP1; MC4R* |
| 86 | rs12454712 | T | C | 0.058269 | *BCL2* |
| 87 | rs10422861 | C | T | 0.058269 | *PEPD* |
| 88 | rs73085586 | G | A | 0.039221 | *LOC284788; LINC00261* |
| 89 | rs6021276 | T | C | 0.039221 | *NFATC2* |
| 90 | rs28637892 | T | G | 0.04879 | *ATXN10; WNT7B* |

SNP, single nucleotide polymorphism.
